# The Transactions of the American Medical Association

**Published:** 1854-12

**Authors:** 


Art. VIII.—*The Transactions of the American Medical Association.* Vol. VII. New York: Charles B. Norton. 1854. pp. 668, 8 vo.

---

We have here another portly volume added to the Transactions of our Medical Congress—a volume comprising a great multitude of subjects, elaborate reports, histories, biographical sketches, essays, and addresses. The appearance of the volume is good, and in point of accuracy, we have reason to believe that it will compare favorably with any preceding number of the series. We can do little more, at present, than give a catalogue of the subjects embraced in the volume.

After the minutes of the meeting, follows the sensible and pertinent address of the Vice-President, Dr. Usher Parsons, an abstract of which we have already laid before our readers. Next to this comes the report of the Committee on *Medical Education*, that prolific, vexed question which has been so often before the Association, and which seems as far as ever from being settled to the satisfaction of many of the members. The renewal of the discussion, from year to year, is attended with good results; it shows that we are not satisfied with our present attainments; it points out defects, and suggests improvements. The present report, by the Chairman of the Committee, J. L. Cabell, M. D., is written with ability, and closes with several resolutions, to the effect, that the views heretofore expressed by the Committees in regard to preliminary education, the extension of the lecture term, &c., be re-affirmed; that "private schools" be recommended; that daily examinations by each Professor are essential to the "active, practical discipline" of the pupils; that medical jurisprudence ought to be included in the curriculum of every medical school; that other modes should be added to the present ones of examining candidates for the doctorate; and finally, that there are "auspicious omens of future progress in the already improved character of our medical literature, and in the evidences of an increasing desire, on the part of a respectable number of medical students, for a higher grade of medical education; and that, in view of such encouraging signs, the Association cherishes an abiding conviction that more thorough and general reforms will be ultimately, though gradually accomplished."

To this report succeeds a report marked by industry and care, by Drs. SUTTON, HASKINS, LIPSCOMBE, and RAMSEY, on the epidemics of Kentucky and Tennessee. This is followed by a paper on Erysipelas, by R. S. HOLMES, M. D. A long and elaborate report on the "medicinal and toxicological properties of the cryptogamic plants of the United States," by F. PAYNE PORCHER, M. D., is the next article. This report was prepared some years since, but its publication was delayed by the absence of the author, who desired to revise its sheets as they passed through the press. It is valuable for the facts presented, and will be a work of reference to those in quest of information on the subject of which it treats.

The Epidemics of Ohio, Indiana, and Michigan are described in the next report, which was prepared by Dr. MENDENHALL, at great length and with great labor. The report is accompanied by a map and meteorological tables. The concluding report, as long and as carefully prepared as any of the preceding, is from the pen of Dr. FENNER. It embraces the epidemics of Louisiana, Mississippi, Arkansas, and Texas. These reports on epidemics possess a permanent value; they are repositories of facts to which the future medical historian will refer with interest. But more than this; they are histories of current events in which all practitioners have a concern. They describe diseases as modified by the various climates and soils of our country, and as affected by the different modes of treatment. It is to be regretted that these instructive reports are not more read.

The *Prize Essay* of Prof. BRAINARD, on a *new method of treating ununited fractures* is published with illustrations. An interesting account of the railroad disaster, at Norwalk, with biographical sketches of those members of the Association who lost their lives on that occasion, follows, and this, with a short paper, by Prof. LINTON, on the identity of bilious and yellow fevers, a report of the Committee on Publication, the catalogue of members, and a full index, completes the volume.

